# From implicit to explicit: an evidence-informed deliberative process for health benefits package revision using the WHO UHC Compendium in Kyrgyzstan

**DOI:** 10.1136/bmjgh-2026-024777

**Published:** 2026-06-26

**Authors:** Gavin Surgey, Gerard Joseph Abou Jaoude, Baktygul Isaeva, Saltanat Zhetibaeva, Mohini Kak, Jyldyz Turgunbaeva, Hassan Haghparast-Bidgoli, Klara Oskombaeva, Rob M P M Baltussen

**Affiliations:** 1 Radboudumc, Nijmegen, Netherlands; 2 Health Economics and HIV and AIDS Research Division, Durban, South Africa; 3 Institute for Global Health, University College London, London, UK; 4 Independent, Bishkek, Kyrgyzstan; 5 World Bank, Vienna, Austria; 6 World Bank Group, Bishkek, Kyrgyzstan; 7 Ministry of Health of the Kyrgyz Republic, Bishkek, Kyrgyzstan; 8 Department for Health Evidence, Radboudumc, Nijmegen, Netherlands

**Keywords:** Health economics, Health policy, Universal Health Care, Decision Making, Health insurance

## Abstract

**Background:**

Translating universal health coverage (UHC) commitments into explicit, evidence-informed benefits packages remains a challenge for many resource-constrained health systems. This paper describes the methods and findings of a full-system benefits package revision applying the WHO Universal Health Coverage Compendium (UHCC) within an evidence-informed deliberative process (EDP) to redesign the State-Guaranteed Benefits Programme in Kyrgyzstan.

**Methods:**

The revision was carried out using the UHCC, following an EDP. The UHCC provided standardised service definitions and resource requirements for analysis. An assessment and appraisal process incorporated technical analysis and governance oversight through the Health Policy Council. The costing methodology estimated input costs per service and multiple financing scenarios were developed with decision-makers to explore coverage–cost trade-offs.

**Results:**

The revision was conducted over 2023–2025, with the Health Policy Council endorsing key methodological decisions. Technical evidence on cost, impact and equity was generated for all services. Through deliberative appraisal, 182 services were prioritised as essential. With an available fiscal space of US$19 per capita, only 66 essential services could be financed under fully public funding. A mixed public–private financing scenario maintained comprehensive essential service coverage while reducing public costs.

**Conclusion:**

Kyrgyzstan’s experience demonstrates that comprehensive, evidence-informed revision of benefits package is feasible in resource-constrained settings when supported by political commitment, strong governance, global tools, structured deliberation and integrated costing. Use of the WHO UHCC enabled standardised service definitions. The approach offers practical lessons for countries seeking to institutionalise priority setting and strengthen progress towards UHC.

**WHAT IS ALREADY KNOWN ON THIS TOPIC**

**WHAT THIS STUDY ADDS**

- This paper presents the first documented application of the WHO Universal Health Coverage Compendium (UHCC) to a complete, system-wide health benefits package revision, demonstrating its feasibility as a practical tool for benefits package design in a resource-constrained LMIC setting.
- Structured deliberation across 424, 182 identified services as high priority. With only US$19 per capita available, a mixed public–private financing scenario was modelled to sustain coverage at reduced public cost.

**HOW THIS STUDY MIGHT AFFECT RESEARCH, PRACTICE OR POLICY**

- Countries with limited Health Technology Assessment capacity can systematically revise benefits packages using the WHO UHCC combined with evidence-informed deliberative processes—this study offers a replicable model for other settings pursuing universal health coverage (UHC).
- Institutionalising explicit benefits package revision, with structured governance, service-level costing and multi-stakeholder deliberation, strengthens accountability in health resource allocation and can accelerate progress towards UHC in countries transitioning away from broadly implicit benefit entitlements.

## Background

In the Kyrgyz Republic, the design and implementation of an explicit health benefits package is recognised as essential for the government’s efforts to achieve universal health coverage (UHC) and improve the quality and sustainability of health services. This priority is underscored in national policy documents, including the Government’s Programme for Public Health Protection and Health Care System Development for 2019–2030 (SPHD2030), which emphasises ‘Healthy Person—Prosperous Country’ and positions investment in health benefits package design as central to national development.^1 2^

An early step in defining a benefits package was the introduction of the State Guaranteed Benefits Package (SGBP) in 2001.^1^ The Mandatory Health Insurance Fund (MHIF) gradually assumed responsibility for administering the SGBP. Following reform in 2006, the MHIF became the national single public payer for SGBP services and the compulsory health insurance system, managing coverage entitlements, copayment structures and purchasing arrangements.^3^ The MHIF also oversees the Additional Drug Package (ADP), which partially subsidises outpatient medicines through user-fee and copayment structures aimed at reducing out-of-pocket (OOP) payments while maintaining a level of cost-sharing.

However, the SGBP has been broadly defined, setting general conditions by level of care and a limited number of diagnostic tests and medications, rather than a systematically defined list of entitlements.^1^ This lack of clarity constrains progress towards UHC by contributing to misaligned budgets, inconsistent access and quality of care, a sizeable uninsured population and persistently high OOP payments. OOP payments account for 38% of total health expenditure, a burden that falls disproportionately on the 31% of people who remain uninsured and must rely on direct payments in the absence of coverage.^1 2^

Recognising these challenges, Kyrgyzstan identified the revision of the SGBP as a priority under SPHD2030. The Ministry of Health (MOH) and the MHIF undertook a complete revision of the SGBP from April 2023 to November 2025, using an evidence-informed deliberative process (EDP).^4 5^ The WHO Universal Health Coverage Compendium (UHCC) served as the core framework for defining services. The UHCC is a comprehensive database that includes accompanying evidence and implementation requirements.^6 7^ It was selected because it provides standardised definitions that ensure comprehensive service coverage while enabling systematic evidence comparison, supporting countries in moving beyond ad hoc package design towards transparent, structured decision-making. This application represents the first use of the UHCC for a complete, system-wide benefits package revision in any country.

This paper describes the stepwise process and methods used to revise the SGBP and examines how explicit benefits package design can be implemented in practice by integrating structured deliberation, service-level evidence and fiscal analysis within existing health sector governance.

## Methods

### Institutional context

The SGBP revision was undertaken as part of the Primary Health Care Quality Improvement Programme (2020–2027), a government initiative co-financed by the World Bank, Swiss Development Agency (SDC) and KfW. This programme provided the institutional and financial framework for conducting a systematic, evidence-informed redesign of Kyrgyzstan’s health benefits package to better align resource allocation with national health priorities.

The technical work was led by a dedicated SGBP Revision Unit established within the MOH and MHIF, with technical support from international consultants based at Radboudumc and University College London, alongside development partners including the World Bank and WHO. Technical working groups (TWGs) were established across key clinical areas to support evidence generation and service mapping activities. Final recommendations were overseen by the Health Policy Council (HPC), a high-level multi-sectoral body responsible for endorsing methodological decisions and submitting approved recommendations to the Cabinet of Ministers, which holds final executive authority for policy adoption.

### Conceptual framework and tools

**Figure 1:**
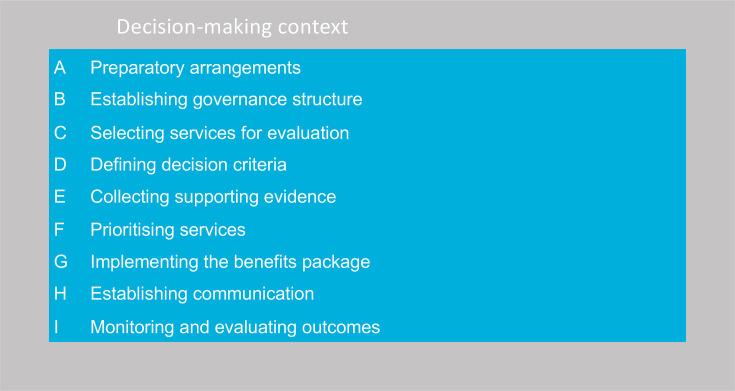
Steps within an evidence-informed deliberative process.

An EDP framework was used to guide the SGBP revision.^4 5 8^ Drawing on international practices in Health Technology Assessment (HTA), the framework is designed to be adaptable to existing institutional arrangements. It provides a stepwise methodology for organising the whole sequence of activities required for benefits package revision. These steps together ensure that the resulting package is both evidence-informed and procedurally legitimate. This paper focuses on the first six steps of the EDP, outlined in figure 1. Steps relating to implementation planning and monitoring fall outside the scope of this analysis.

The WHO UHCC was used as a practical tool to structure and organise services considered for inclusion in the revised SGBP.^6 7 9^ Existing services were mapped by the TWGs to the Compendium to create a complete and standardised inventory of services across levels of care, including community, primary, outpatient and inpatient services.

To operationalise the UHCC for analysis, service design and costing, the Service Package Delivery & Implementation (SPDI) platform was used. Built directly on the UHCC database, SPDI provides a structured, nested architecture aligned with WHO guidelines and expert consensus.^6 7 9^ The platform supports a systematic assessment of existing service delivery and offers an interactive environment for benefits package design. It includes detailed information on health workforce requirements, products and technologies, delivery platforms and disease burden for more than 500 services, enabling integrated planning and implementation. SPDI ensured consistent use of UHCC data across service mapping, prioritisation and costing activities.

## Results

### A: Preparatory arrangements

The preparatory phase established the methodological foundations for the SGBP revision, with key decisions on analytical scope and tools documented in the Methods section.

### B: Establishing governance structure

**Figure 2:**
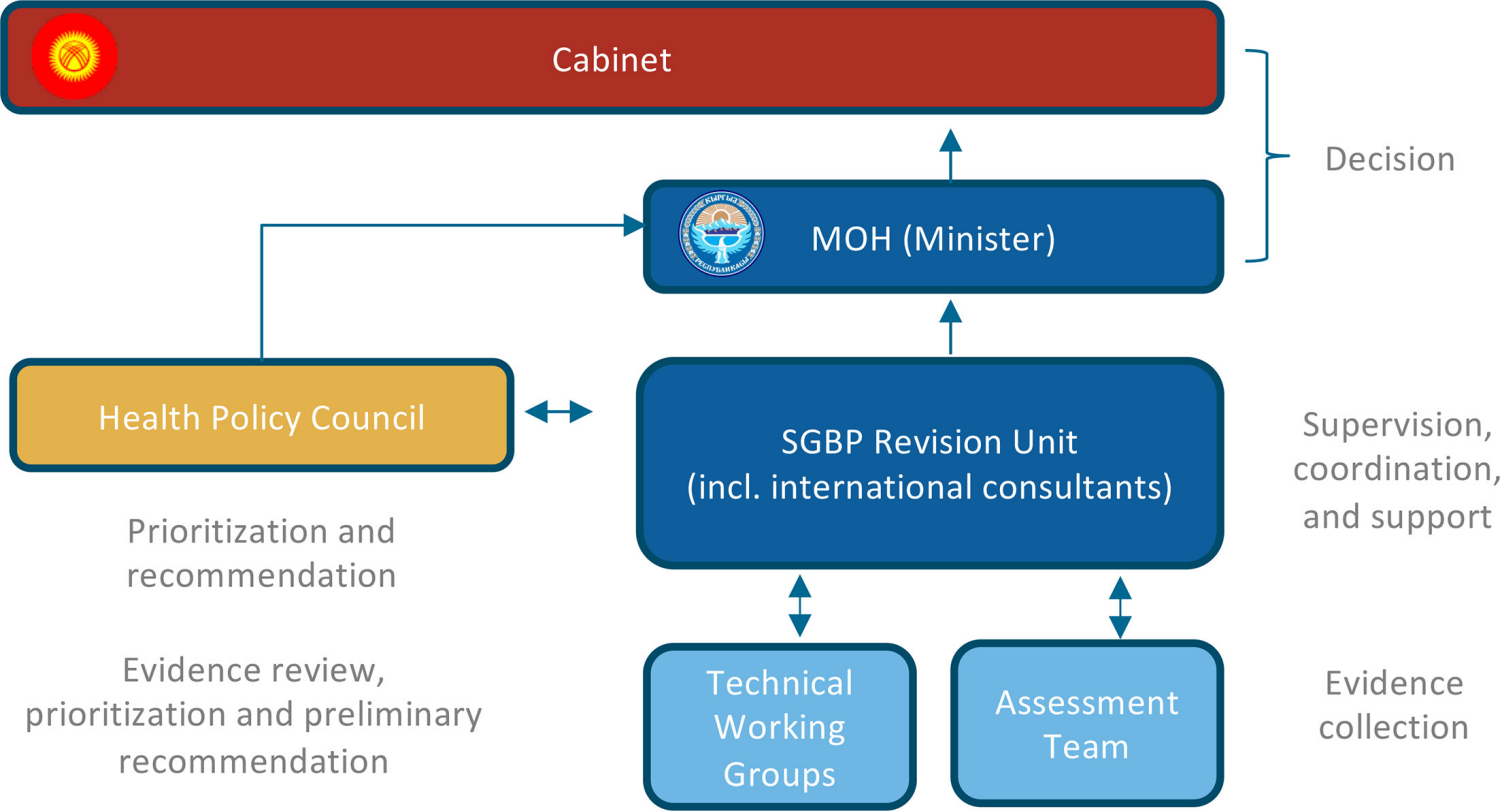
SGBP revision governance structure. MOH, Ministry of Health; SGBP, State-Guaranteed Benefits Programme.

A multi-stakeholder governance structure was established in mid-2023, as shown in figure 2.

The TWGs were organised into disease-specific and thematic areas: child health, maternal health, non-communicable diseases, communicable diseases and injuries (including foundational services). TWG members underwent comprehensive training on the EDP process, UHCC framework, evidence assessment methods and deliberative priority-setting. These groups conducted evidence review, prioritisation and developed preliminary recommendations within their respective areas of expertise, which were then forwarded to the HPC. The Assessment Team, comprising members drawn from the TWGs, contributed to data collection and validation across thematic areas. The SGBP Revision Unit supported the TWGs and Assessment Team and ensured methodological consistency across all steps of the process.

### C: Selecting services for evaluation

Service selection took place in the second half of 2023. During this step, a comprehensive inventory of services for assessment was defined by systematically identifying both existing health services currently provided in Kyrgyzstan and potential services not yet available but under consideration for inclusion. All services were mapped to the WHO UHCC to establish the full set of services eligible for prioritisation under the revised SGBP.

TWGs reviewed and validated detailed service-level inputs, including personnel requirements, annual visit frequencies, medicine specifications, diagnostic requirements, consumables and population-in-need estimates. Through this process, the TWGs collectively developed an aspirational list of 424 services, encompassing both currently delivered services and services considered for future inclusion.

### D: Defining decision criteria

A workshop in October 2023 brought together 62 stakeholders to define criteria for service prioritisation. Based on deliberation around several case studies and drawing on international frameworks, participants identified eight priority criteria: clinical effectiveness, cost-effectiveness, life-threatening conditions, burden of disease, financial risk protection, budget impact and equity considerations related to both disease severity and vulnerable populations—particularly pregnant women and children (online supplemental material 1). Disease severity was defined as the extent to which a condition impacts health, including mortality and morbidity effects.

Details on the workshop on decision criteria, including the process and criteria definitions, are provided in online supplemental material 1.

### E: Collecting supporting evidence

Evidence was compiled from local, regional and global sources and synthesised against the agreed decision criteria by the Assessment Team, in collaboration with the SGBP Revision Unit. Evidence scores for all 182 services are reported in online supplemental material 2, online tables S1 and S2.

For cost-effectiveness, the team relied primarily on average cost-effectiveness ratios (ACERs) from the WHO-CHOICE (WHO Choosing Interventions that are Cost-Effective) database and Disease Control Priorities 3rd Edition (DCP3),^10^ which are linked to UHCC services,^9^ supplemented where necessary by regional literature and peer-reviewed studies. WHO-CHOICE ACERs represent the ratio of intervention costs to health benefits relative to a ‘null’ scenario without intervention, measured in disability-adjusted life years (DALYs) averted, accounting for both morbidity and mortality reduction.

Clinical effectiveness was not quantified since all services mapped to the WHO UHCC were considered interventions with established effectiveness.^11^ Disease severity and equity considerations were qualitatively assessed based on expert opinion from TWG members.^11^

Diseases were categorised by severity (very severe, moderately severe, not severe) based on expert opinion. Priority groups were identified through expert consensus during deliberations, with special consideration given to pregnant women, children and other vulnerable populations.

### Estimating costs

The costing methodology was formally endorsed by the HPC in early 2024, after which service-level costing was undertaken alongside the prioritisation process. A comprehensive costing model was developed and applied using the WHO UHCC architecture to estimate annual costs for each of the 424 services across Kyrgyzstan’s health system delivery levels.^12^ For each service, the model specified a visit type, the population requiring the service (population in need), the types of health workers needed, the estimated time and related health products.

Unit costs were derived from multiple sources: medicine costs from Kyrgyzstan’s national price list (supplemented by Uzbekistan data when unavailable), diagnostic costs from literature review and consumables/facility overheads from local costing studies using percentage mark-ups. Population-in-need estimates utilised over 600 data points from sources prioritised for geographic relevance: Kyrgyzstan-specific data, regional estimates, programme-specific models and literature reviews.

Full details of the cost model are reported in Jaoude *et al*.^12^

### Defining fiscal space

Available fiscal space was determined through a line-by-line review of the 2025 MOH and MHIF budgets. A key decision was made to focus the prioritisation analysis on fungible budget items—those that could be reallocated across services based on prioritisation decisions. Personnel salaries and national-level administrative costs were therefore excluded from the available fiscal space, as health worker salaries are largely fixed within the current system and certain system-level functions are required regardless of which services are included in the SGBP. This approach focused the fiscal analysis on non-personnel inputs (medicines, diagnostics, consumables and facility-level operational costs), which represent the areas where prioritisation decisions could most directly influence resource allocation. The analysis resulted in US$19 per capita in available fiscal space for reallocation.

It should be noted that while the fiscal space analysis focused on non-personnel costs, the comprehensive costing model (described earlier) included both personnel and non-personnel requirements for all services to support a full understanding of implementation needs.

### F: Prioritising services

Prioritisation was carried out sequentially, with primary healthcare services prioritised first in early to mid-2024, followed by inpatient and hospital-based services in late 2024. The prioritisation process addressed the challenge of a list of 424 aspirational health services with an estimated annual non-personnel cost of US$139 per capita, exceeding available fiscal space of US$19 per capita by US$120 per capita. A three-stage methodology systematically determined service inclusion based on evidence review, multi-criteria analysis and fiscal constraints, with policy scenarios developed to present decision-makers with alternative service coverage-cost trade-offs.

Stage 1 automatically included 86 services based on established international evidence and foundational importance to health system functioning. Services qualified through either (1) being classified as foundational services essential for basic health system operation (basic emergency care, essential childbirth services, routine immunisation) or (2) being exceptionally cost-effective (ACER<US$100 per DALY averted). The identification process drew on DCP3 Health Priority Package,^10^ WHO-CHOICE cost-effectiveness analyses^13^,^16^ and WHO ‘best buys’ for non-communicable disease prevention—systematic evidence reviews by international expert panels informing benefits package design across multiple low- and middle-income countries.^17^

Stage 2 applied systematic evidence-informed prioritisation to the remaining 338 services through structured TWG deliberations across the defined decision criteria (online supplemental table S3, online supplemental file 1). Facilitators guided TWG members through a deliberative process that involved reviewing the UHCC service, presenting evidence across all decision criteria and engaging in structured argumentation to reach a unanimous agreement on priority classification (high, medium or low). TWGs interpreted evidence on all eight decision criteria (online supplementary material 1) during a workshop, with each TWG covering between 8 and 80 services within their expertise area. This resulted in 182 services classified as high priority, 135 as medium priority and 107 as low priority (online supplemental material 2, online table S1).

Stage three assessed fiscal feasibility (online supplemental material 1, online table S4) by identifying which services could be included within realistic budget constraints. This stage aligned the prioritised services with currently available fiscal space, ensuring that the final package reflected implementable coverage-cost trade-offs rather than a theoretical list of desired services. On this basis, three policy scenarios were developed to illustrate alternative coverage and financing responses, ranging from strict adherence to the current fiscal envelope to options requiring additional resources.

Cost estimates for each service and a summary of services by scenario and programme area are reported in online supplemental material 2, online table S1–S3.

### Scenario options for decision-making: modelling the cost and impact of policy options

Three policy scenarios were modelled to illustrate alternative coverage-cost trade-offs (table 1). The ‘universal coverage’ scenario represents the scenario with maximum health impact by fully subsidising all 182 highest-priority services at an estimated annual cost of US$53.76 per capita for non-personnel inputs, thereby exceeding the current fiscal space of US$19 per capita by approximately US$35 per capita. This scenario would avert approximately 10.1 million DALYs.

**Table 1:** Summary of policy options modelled across three policy scenarios

Scenario	Services included	Cost per capita (US$)	Health impact	Public funding structure	Patient payment structure	Services not in package
Scenario 1: Universal public coverage	182 high-priority services	53.76	10.10 million DALYs averted	100% publicly funded by MOH/MHIF	0%—no patient payments	100% OOP for 242 excluded services
Scenario 2: Budget-constrained coverage	66 highest-priority services	21.16	7.58 million DALYs averted (–2.52 million vs scenario 1)	100% publicly funded by MOH/MHIF	0%—no patient payments	100% OOP for 358 excluded services
Scenario 3: Mixed public–private financing	182 high-priority services	47.74	10.09 million DALYs averted (–10 000 vs scenario 1)	151 services: 100% public funding 31 services: ADP reimbursement rates	151 services: 0% patient cost 31 services: patient co-payments per ADP rates	100% OOP for 242 excluded services

* Service-level detail by scenario is in online supplementary file 2, online table 3.

ADP, Additional Drug Package; DALYs, disability-adjusted life years; MHIF, Mandatory Health Insurance Fund; MOH, Ministry of Health; OOP, out-of-pocket; SGBP, State Guaranteed Benefits Package.

The ‘budget-constrained coverage’ scenario required further prioritisation of the 182 highest-priority services to identify services feasible within available fiscal space. TWGs conducted an additional prioritisation exercise, systematically reviewing the 182 services to select a subset of 66 services deemed most critical. This constrained package required US$21.16 per capita annually for non-personnel inputs, above the available fiscal space of US$19 per capita. Compared with the universal coverage scenario (which averts approximately 10.1 million DALYs), this approach averts 7.6 million DALYs, implying a reduction of 2.5 million DALYs. The scenario excludes critical interventions across major disease areas (including diabetes, hypertension, mental health, HIV/AIDS, tuberculosis, hepatitis, tetanus and heart failure), reproductive health services and emergency services. The exclusions conflict with government commitments and national health priorities, rendering this scenario politically unacceptable.

The ‘mixed public–private financing’ scenario retains coverage of all 182 high-priority services but differentiates financing arrangements across them. It applies existing MHIF entitlements and ADP reimbursement rates to 31 services alongside full public funding for 151 services. This would cost the MOH and MHIF an estimated US$47.74 per capita annually for non-personnel inputs, which exceeds the current fiscal space of US$19 per capita but represents a reduction of approximately US$6 per capita relative to the ‘universal coverage scenario’. Compared with the ‘universal coverage scenario’, this approach would result in similar levels of DALYs averted.

The prioritisation phase was concluded with final policy scenarios under review through an inter-ministerial process since November 2025. Given the substantial increase in per capita investment required, the final decision will depend on the advocacy and political mobilisation efforts by the MOH.

Read together, the three scenarios define a phased pathway: the budget-constrained package as a near-term coverage floor, the mixed public–private arrangement as a fiscally achievable intermediate step and universal coverage as the longer-term aspiration towards which incremental budget commitments can be directed. This phased interpretation avoids creating an explicit package that is unaffordable in practice, while preserving the 182-service list as a transparent medium-term benchmark for progressive expansion.

## Discussion

This study represents the first application of the WHO UHCC framework to redesign a health system-wide benefits package through an EDP approach. The Kyrgyzstan experience demonstrates how standardised service definitions, systematic data collection and validation, service-level costing and structured stakeholder deliberation can translate UHC commitments into implementable packages.

The analytical approach revealed a substantial gap between population health needs, policy commitments and current public financing. While 182 services were identified as high priority from 424 evaluated, only 66 can be financed within the existing budget envelope (online supplemental file 2 and online supplemental tables S1 and S3), excluding essential services for major non-communicable diseases, infectious diseases, mental health and emergency care. Meaningful progress towards UHC will require increased public financing and closer alignment between policy ambitions and fiscal capacity.

The mixed public–private financing scenario offers a middle path, retaining all 182 high-priority services through differentiated financing arrangements. This exceeds the current fiscal envelope by US$29 per capita, with the financing gap under discussion between the MOH and Ministry of Finance. Read together, the three scenarios define a phased pathway: the budget-constrained package as a near-term coverage floor, the mixed public–private arrangement as a fiscally achievable intermediate step and universal coverage as the longer-term aspiration towards which incremental budget commitments can be directed.

The process was resource-intensive, requiring sustained investment over approximately 2 years in technical expertise, stakeholder engagement and training. Comparing this cost against alternative approaches to benefits package revision is beyond the scope of this study and an important gap for future research. What the exercise produced, however, was not only a revised package. It generated durable institutional outputs: a trained cadre of experts familiar with prioritisation and, importantly, a service-level costing model available for future updates. Subsequent revision cycles should be substantially less costly, as these foundations are now in place. Against this, the status quo carried its own costs: a broadly defined, implicit package associated with 38% OOP expenditure and persistent inequities in access

This study demonstrates that a full-system benefits package revision using the UHCC within an EDP can be implemented in a resource-constrained setting. It does not demonstrate that this approach is superior to alternative methods, such as expert-led prioritisation, adaptation of existing global priority lists or technical costing of existing entitlements. Such comparisons would require a different study design. The approach to defining services using standardised UHCC parameters (target populations, delivery platforms, human resources, diagnostics and pharmaceuticals), while engaging stakeholders through structured deliberation, ensured that prioritised services reflected both technical evidence and societal values. The costing exercise also proved influential beyond its technical purpose, framing discussions with senior policymakers in concrete terms. Demonstrating substantial health returns shifted discussions from affordability towards value and opportunity cost, reinforcing that underinvestment carries substantial costs in avoidable health losses.

### Implementation experience and lessons learned

Several enabling conditions were essential to this process. A formal institutional mandate through the Primary Health Care Quality Improvement Programme provided the authority to commission the revision. A dedicated Revision Unit, established within the MOH and MHIF, ensured methodological continuity across changes in senior leadership. International facilitation expertise was required for the deliberative components: the criteria selection workshop, TWG training and structured prioritisation sessions. Countries without access to such expertise may need to invest in domestic facilitation capacity or partner with regional technical institutions before undertaking a full-system revision.

Successful application required significant adaptation to the local context. TWG members systematically reviewed and adapted delivery models to reflect local practice patterns, reimbursement protocols and clinical guidelines. While resource-intensive, this contextualisation ensured cost estimates reflected local realities, enhanced stakeholder buy-in and facilitated implementation. While international frameworks provide methodological rigour, meaningful implementation depends on local adaptation.

Several design choices proved important in practice. During the criteria selection workshop, individual scoring via digital voting preceded group discussion. This prevented premature consensus around dominant voices, revealed the distribution of individual values and gave facilitators a structured basis for guiding deliberation. TWG training used examples to make abstract criteria operationally meaningful; participants reached shared understanding of criteria definitions faster when trade-offs were framed around familiar services.

Data availability posed significant challenges, compounded by multiple decision criteria requiring related evidence. Where local evidence was not readily available, the team used regional proxies and expert elicitation with clinical specialists, though this proved resource-intensive. The qualitative elicitation process captured stakeholder values but proved resource-intensive for TWG members. Some quantitative criteria, like financial risk protection, were difficult to understand and largely duplicated information already captured in cost and budget impact analyses. Deliberations revealed final decisions rested heavily on cost-effectiveness, disease burden and budget impact, while other criteria played secondary roles. Future updates might simplify the criteria, decreasing the data collection effort and reducing the burden on experts.

Those undertaking similar exercises should anticipate several operational risks. Leadership turnover within the MOH mid-process required re-engagement of incoming officials who had not been part of earlier consensus-building, a time cost not anticipated at the outset. This underscores the importance of documenting methodological decisions formally rather than relying on institutional memory. Sustaining TWG participation over a 30-month process also proved challenging, with stakeholders facing competing demands. Finally, communicating the fiscal envelope to participants from the start is essential. Introducing budget constraints late, after TWGs have invested in services that are subsequently excluded on cost grounds, generates frustration that is difficult to reverse.

Technical limitations also need recognition. The costing analysis assumed existing workforce capacity would suffice without assessing whether staffing levels, skill mix or geographic distribution could accommodate increased demand. Variations in quality of care, quality assurance mechanisms and the role of private providers (particularly where informal payments predominate) were not comprehensively assessed. The analysis presented full implementation scenarios without phased rollout strategies; practical implementation will require sequencing decisions about service prioritisation and geographic phasing, likely undertaken in the next phase. This study did not establish institutional mechanisms for ongoing evidence review, periodic updates and new technology assessment, which are needed to sustain an evidence-informed approach; future workstreams will need to focus on these sustainability mechanisms alongside facility readiness and information systems.

Overall, this study illustrates how countries can move from implicit to explicit benefits packages through a structured, evidence-informed process, even under severe fiscal constraints. By documenting the full sequence from service identification and prioritisation to costing and policy scenario development, the Kyrgyzstan experience offers practical and transferable lessons for low- and middle-income countries seeking to strengthen benefits package design, particularly those without established health technology assessment systems. The approach demonstrates how global public goods such as the UHCC can be combined with national deliberative processes to support more transparent, coherent and implementable decisions on health service coverage.

## Supplementary material

10.1136/bmjgh-2026-024777 online supplemental file 1

10.1136/bmjgh-2026-024777 online supplemental file 2

10.1136/bmjgh-2026-024777 online supplemental file 3

## Data Availability

All data relevant to the study are included in the article or uploaded as supplementary information.
