# Supplementary material for: From implicit to explicit: an evidence-informed deliberative process for health benefits package revision using the WHO UHC Compendium in Kyrgyzstan
Source: BMJ Glob Health. 2026 Jun 26;11(6):e024777. doi: 10.1136/bmjgh-2026-024777 (PMC13311575; doi:10.1136/bmjgh-2026-024777)
Supplement: online supplemental file 2 [file bmjgh-11-6-s002.docx]

### BMJ Global Health Author Reflexivity Statement

Adapted from Morton, B., Vercueil, A., Masekela, R., Heinz, E., Reimer, L., Saleh, S., Kalinga, C., Seekles, M., Biccard, B., Chakaya, J., Abimbola, S., Obasi, A. and Oriyo, N. (2022), Consensus statement on measures to promote equitable authorship in the publication of research from international partnerships. Anaesthesia, 77: 264-276. <https://doi.org/10.1111/anae.15597>

| **Study conceptualisation** | |
| --- | --- |
| 1. How does this study address local research and policy priorities? | This study directly responds to Kyrgyzstan's national health financing reform agenda. Since 2011, the country has operated a State-Guaranteed Benefits Package (SGBP) that defines service entitlements only in broad categorical terms, making it impossible to cost, plan, or enforce. The Ministry of Health and the Mandatory Health Insurance Fund (MHIF) identified SGBP revision as a priority under ongoing UHC reforms. This work provides the fiscal analysis, service prioritisation, and policy trade-off modelling that Kyrgyz policymakers need to make decisions about the revised package. |
| 1. How were local researchers involved in study design? | The study design was shaped through an Evidence-informed Deliberative Process (EDP) governance structure established in collaboration with Kyrgyz institutions. An Assessment Team and Costing Team, both comprising MOH and MHIF representatives, co-designed the data collection instruments, service definitions, and prioritisation criteria. Technical Working Groups (made up of national programme managers from areas including mental health and oncology, alongside MHIF staff and development partners) reviewed the analytical approach at each stage. The Council for Health Policy provided oversight and shaped the framing of policy scenarios. |
| **Research management** | |
| 1. How has funding been used to support the local research team(s)? | World Bank funding supported substantive involvement of Kyrgyz government staff throughout the project. Dedicated secretariat capacity was funded within country structures to coordinate TWG meetings and maintain documentation. Training in the use of the WHO SPDI Platform and in multi-criteria decision analysis was delivered to members of the Assessment Team, building institutional capacity that will remain in the country after the project closes. |
| **Data acquisition and analysis** | |
| 1. How are research staff who conducted data collection acknowledged? | Research staff who were significantly involved are listed as authors. members of the technical working groups who supported the validation process are acknowledged as Technical Working Groups Members.  Annex 1 of the final research report lists all members of the Assessment Team, Costing Team, Technical Working Groups, and Council for Health Policy who contributed to data collection and analysis. Programme managers who provided service-level clinical and epidemiological data are credited by name and institution. Translators and data administrators who supported the process are acknowledged in the report. |
| 1. How have members of the research partnership been provided with access to study data? | All evidence summaries generated for the 424 services - covering clinical effectiveness, cost-effectiveness, burden of disease, equity, and cost data - were shared with Country TWG members before prioritisation workshops. The WHO SPDI Platform, used to define and cost services, is accessible to MOH and MHIF staff. Data on fiscal space, budget scenarios, and costing assumptions were shared with the Council for Health Policy as part of the deliberative process. The full dataset and model underpinning the scenario analysis have been provided to the MoH for ongoing use. The country continues to make use of the cost model to refine service selection. |
| 1. How were data used to develop analytical skills within the partnership? | Structured training sessions on criteria, fiscal space assessment, and service costing were delivered to members of the Assessment and Costing Teams. Working through the prioritisation of 424 services in TWG sessions and applying eight decision criteria to real country data, provided applied learning that reinforced conceptual training. The costing model was built collaboratively, with Kyrgyz team members contributing local unit cost data and validating assumptions, giving them working knowledge of the methodology rather than passive familiarity with outputs. The country continues to make use of the cost model to refine service selection. |
| **Data interpretation** | |
| 1. How have research partners collaborated in interpreting study data? | Data interpretation was distributed across the governance structure. TWGs reviewed and deliberated over evidence for each service in their clinical area, reaching consensus on priority levels before results moved to the Council for Health Policy. The Council reviewed prioritisation outputs, challenged assumptions, and approved the final list of 177 highest-priority services. Scenario modelling of budget trade-offs was presented to country stakeholders in draft form, with Kyrgyz team members raising questions about assumptions that led to revisions in the analysis. Final interpretation of what the data mean for policy was the responsibility of Kyrgyz decision-makers, not the international consultants. |
| **Drafting and revising for intellectual content** | |
| 1. How were research partners supported to develop writing skills? | Kyrgyz team members contributed to drafting sections of the technical report, particularly those covering the governance process, local data sources, and policy context. Iterative feedback was provided by UCL and Radboudumc co-authors on structure and clarity. The documentation of TWG deliberations - which Kyrgyz secretariat staff prepared - forms a substantial part of the evidence base for the report and required sustained analytical writing. |
| 1. How will research products be shared to address local needs? | The final report is submitted directly to the Ministry of Health and the Cabinet of Ministers to inform a decision on SGBP revision. A policy brief summarising key findings and trade-offs has been prepared for decision-makers who require accessible rather than technical outputs. All materials are available in Russian, the primary working language of government in Kyrgyzstan, as well as English. Country stakeholders retain ownership of the data and the models. The country continues to make use of the cost model to refine service selection. |
| **Authorship** | |
| 1. How is the leadership, contribution and ownership of this work by LMIC researchers recognised within the authorship? | The authorship team includes Kyrgyz researchers and government officials from MOH and MHIF who contributed to the governance structure, data collection, and deliberative process. The ordering of authors reflects contribution, with Kyrgyz partners holding named authorship positions rather than being listed solely in acknowledgements. Ownership of the policy product rests with the Government of Kyrgyzstan. |
| 1. How have early career researchers across the partnership been included within the authorship team? | Early career researchers from Kyrgyzstan contributed to data collection and analysis as they formed a part of the country secretariat. They were included in the authorship. Mentorship on the process was provided by senior members of the UCL and Radboudumc teams. |
| 1. How has gender balance been addressed within the authorship? | The authorship includes both male and female researchers from Kyrgyzstan and the international partner institutions. |
| **Training** | |
| 1. How has the project contributed to training of LMIC researchers? | The project delivered applied training in EDP methodology, multi-criteria decision analysis, fiscal space assessment, and health service costing to Kyrgyz government staff. Participation in TWG sessions and Council deliberations also built skills in evidence appraisal and structured decision-making. The project team has documented the full process, creating a reference resource for future benefits package revision cycles in Kyrgyzstan and potentially for other countries in the region. |
| **Infrastructure** | |
| 1. How has the project contributed to improvements in local infrastructure? | The project established a functioning governance architecture for health benefits package decision-making that did not previously exist in this form in Kyrgyzstan. The Assessment Team and Costing Team represent standing institutional capacity within MOH and MHIF. All data, models, and service definitions developed during the project are held by Kyrgyz institutions. The WHO SPDI Platform has been adopted as the standard service definition and costing tool, providing a durable framework for future revision work. The country continues to make use of the cost model to refine service selection and this is in use as of April 2026. |
| **Governance** | |
| 1. What safeguarding procedures were used to protect local study participants and researchers? | This study did not involve primary data collection from patients or members of the public. Data used in the analysis were drawn from routine government administrative sources (MHIF budget reports, MOH service statistics, national medicines price lists) and published literature. All Kyrgyz government staff who participated did so in their professional capacity, with the knowledge of their institutions. No individual-level personal data were collected or processed. The project operated within the governance and oversight framework of the Ministry of Health and under World Bank fiduciary and safeguarding policies. |
