# Supplementary material for: From implicit to explicit: an evidence-informed deliberative process for health benefits package revision using the WHO UHC Compendium in Kyrgyzstan
Source: BMJ Glob Health. 2026 Jun 26;11(6):e024777. doi: 10.1136/bmjgh-2026-024777 (PMC13311575; doi:10.1136/bmjgh-2026-024777)
Supplement: online supplemental file 3 [file bmjgh-11-6-s003.docx]

# Supplementary File 1. Decision Criteria for Prioritisation of Health Services in Kyrgyzstan’s State-Guaranteed Benefits Programme

# 1. Introduction

This supplement details the process by which eight decision criteria were identified and defined for use in the revision of Kyrgyzstan's State-Guaranteed Benefits Programme (SGBP). It accompanies the main manuscript and corresponds to the criteria selection activities described in Section D of the Methods.

Decision criteria are the explicit values and dimensions of evidence used to compare and rank health services for inclusion in a benefits package. Their selection is itself a deliberative act, it determines what counts as evidence and which objectives the health system prioritises. The criteria adopted in Kyrgyzstan reflect both international best practice in health benefit package design and the specific policy context and values of Kyrgyz stakeholders.

The criteria selection workshop was held on 11 October 2023 as part of a broader five-day seminar (9–13 October 2023) attended by members of the Health Policy Council, Technical Working Group (TWG) members, and Ministry of Health and Mandatory Health Insurance Fund (MHIF) representatives, supported by international consultants.

# 2. Criteria Selection Workshop: Process and Activities

The workshop followed a structured process designed to build shared understanding before moving to formal selection:

**Table S1.** Criteria selection workshop: structured process and activities

| **Step** | **Activity** | **Description** |
| --- | --- | --- |
| 1 | **Establishing shared understanding** | Plenary presentation on what decision criteria are and why they matter, using examples from consumer goods (the 'toaster example') and health services. Facilitated by international consultants (Radboudumc and UCL) with reference to WHO guidance and a review of national health strategy documents. |
| 2 | **Group brainstorming on example interventions** | Participants were introduced to six example interventions (insulin for diabetes, assisted hospital delivery, glasses for children, trastuzumab for breast cancer, smoking cessation, mammogram screening). Small groups identified 3–4 criteria they would use to include or exclude each service. Findings were presented in plenary. |
| 3 | **Synthesis of brainstorm responses** | The project team categorised the criteria raised in group discussions into 11 broad domains: effectiveness (including evidence quality), safety, cost-effectiveness, burden of disease, severity of condition, vulnerable groups, financial burden to patient, budget impact, feasibility, and emergency care. |
| 4 | **Presentation of international criteria frameworks** | Consultants presented WHO-recommended criteria and criteria used in comparable country contexts, enabling participants to compare their brainstormed criteria against established frameworks. |
| 5 | **Individual Mentimeter voting** | All participants independently rated eight candidate criteria on a Likert scale (1 = not important; 5 = very important) and then ranked them in order of priority. This captured individual values anonymously before group deliberation. |
| 6 | **Plenary discussion and finalisation** | Results from the Mentimeter exercise were presented and discussed. The group reached consensus on eight criteria to be used in the SGBP revision, with clarification of definitions and the role of each criterion in the prioritisation process. |

The Mentimeter voting exercise captured individual stakeholder values anonymously before plenary deliberation, reducing the risk of anchoring bias in group discussion. Results were projected in real time and used to structure the final discussion on criteria selection and weighting.

# 3. Stakeholder Voting Results (Mentimeter Exercise)

The table below summarises the results of the individual Likert scoring and ranking exercise. Eight criteria emerged as priority criteria for adoption in the SGBP revision process.

**Table S2.** Stakeholder voting results (Mentimeter exercise): importance ratings and consensus rankings for candidate criteria

| **Criterion** | **Average importance rating (Likert 1–5)** | **Consensus ranking** |
| --- | --- | --- |
| **Clinical effectiveness** | High | 1st |
| **Cost-effectiveness** | High | 2nd |
| **Burden of disease** | High | 3rd |
| **Severity / life-threatening conditions** | High | 4th |
| **Financial risk protection** | High | 5th |
| **Budget impact** | Medium-high | 6th |
| **Equity — vulnerable populations** | Medium-high | 7th |
| **Feasibility** | Medium | Not included as standalone criterion* |

* Feasibility was incorporated as a consideration within Stage 3 of the prioritisation process (fiscal feasibility assessment) rather than as a standalone scoring criterion in TWG deliberations.

# 4. Criteria Definitions, Evidence Bases, and Operationalisation

The following table provides formal definitions for each of the eight adopted criteria, the evidence sources used to populate each criterion, how each criterion was operationalised in the prioritisation process, and notes on practical application.

**Table S3.** Formal definitions, evidence bases, and operationalisation of the eight adopted decision criteria

| **#** | **Criterion** | **Definition** | **Evidence source** | **Operationalisation** | **Notes** |
| --- | --- | --- | --- | --- | --- |
| 1 | **Clinical Effectiveness** | The extent to which a service produces the intended health outcome under real-world conditions. All services mapped to the WHO UHC Compendium were assumed to meet a minimum threshold of clinical effectiveness, as the Compendium includes only evidence-based services. | Services were cross-referenced with the WHO UHC Compendium. Clinical effectiveness was not re-quantified; inclusion in the Compendium served as the evidence base. | Binary confirmation: service is included in the WHO UHC Compendium (Yes/No). Informed Stage 1 automatic inclusion of foundational services. | Deliberately not scored on a continuous scale given Compendium pre-screening. Used as a qualifying criterion rather than a ranking criterion. |
| 2 | **Cost-Effectiveness** | The health benefit generated per unit of cost, assessed relative to Kyrgyzstan's GDP per capita (~USD 1,900). Operationalised using Average Cost-Effectiveness Ratios (ACERs) expressed as cost per DALY averted. | Primary source: WHO-CHOICE (Choosing Interventions that are Cost-Effective) database. Supplemented by Disease Control Priorities (DCP3) data and peer-reviewed literature where WHO-CHOICE data were unavailable. | Three-category classification: Category 1: ACER ≤ 1× GDP per capita (≤ ~USD 1,900) — cost-effective Category 2: ACER 1–3× GDP per capita — moderately cost-effective Category 3: ACER > 3× GDP per capita — less cost-effective 'No CEA available': insufficient evidence | Where no ACER was available, services were assessed qualitatively by TWGs drawing on programme experience and regional literature. Services with ACER < USD 100/DALY averted were classified as exceptionally cost-effective and included in Stage 2 automatic inclusion. |
| 3 | **Burden of Disease** | The magnitude of health loss attributable to a disease or condition in the Kyrgyzstan population, measured in DALYs (Disability-Adjusted Life Years) averted. | Global Burden of Disease (GBD) estimates, national mortality and morbidity data, and MHIF utilisation records. Disease-specific burden estimates were compiled by the Assessment Team for each of the five programme areas. | Quantitative where available: estimated DALYs averted per service at population level. Where estimates were unavailable, TWGs used qualitative assessment based on epidemiological knowledge. | High burden of disease was weighted particularly heavily in TWG deliberations for NCD and infectious disease services. 'No impact estimate available' was recorded for 110 of 182 services due to data limitations. |
| 4 | **Life-Threatening Conditions** | Whether the condition addressed by the service poses a direct risk to life without intervention, including emergency and acute care situations. | Clinical judgement by TWG members and reference to clinical guidelines. Emergency and acute care services were flagged by disease-specific TWGs. | Binary classification applied during TWG deliberations. Services addressing life-threatening conditions received priority consideration in Stage 3 deliberations. | This criterion reinforced the inclusion of emergency obstetric care, acute stroke management, and emergency trauma services even where cost-effectiveness data were limited. |
| 5 | **Financial Risk Protection** | The extent to which public financing of a service protects households from catastrophic out-of-pocket (OOP) expenditure. Assessed at two thresholds commonly used in the literature. | Household expenditure data, MHIF reimbursement records, and national OOP expenditure estimates. Applied to services where cost to the patient without public coverage would be significant. | Two binary flags: FRP (10% threshold): service would impose catastrophic OOP costs at 10% of household income FRP (25% threshold): service would impose catastrophic OOP costs at 25% of household income | Financial risk protection was a particularly salient criterion given that 31% of total health expenditure in Kyrgyzstan is OOP. Services scoring positively on FRP were prioritised for full public financing in Scenario 3. |
| 6 | **Budget Impact** | The total fiscal cost of including a service in the SGBP at full population coverage, expressed as an annual per capita cost (non-personnel inputs only) and as a percentage of the available MOH/MHIF budget. | Comprehensive bottom-up costing model developed using the SPDI (Service Package Delivery & Implementation) platform, based on the WHO UHC Compendium service architecture. Input costs drawn from Kyrgyzstan national price lists, supplemented by Uzbekistan data where gaps existed. | Cost per capita (USD, non-personnel inputs: medicines, diagnostics, consumables, facility-level operational costs). Personnel costs excluded as salaries are largely fixed within the current system. % of available budget = service cost / total MOH+MHIF non-personnel budget | Budget impact was a binding constraint underpinning all three policy scenarios. The total cost of all 182 high-priority services (~USD 54/capita) exceeds current fiscal space (~USD 19/capita) by approximately USD 35/capita. |
| 7 | **Equity: Severity of Disease** | Whether the disease or condition is particularly severe in nature, justifying prioritisation on grounds of need. Captures the principle that those with the greatest health need should receive priority. | Clinical classification by TWGs drawing on ICD-10 categorisations, clinical guidelines, and programme expertise. | Four-category classification applied by TWGs: Very severe Moderately severe Not severe High priority (existing national priority status) | Severity was assessed as a component of equity alongside vulnerability (Criterion 8). Services rated 'very severe' or 'high priority' received additional weight in deliberative scoring. |
| 8 | **Equity: Vulnerable Populations** | Whether a service disproportionately benefits vulnerable population groups, including pregnant women, children, the elderly, people with disabilities, and low-income households. Reflects the equity principle of prioritising the worst-off. | Programme-level epidemiological data and TWG clinical knowledge. Particular attention was paid to maternal and child health services, consistent with existing national exemption policies. | Binary classification: does the service disproportionately benefit a defined vulnerable group? (Yes/No). Specific attention to pregnant women and children as identified by deliberating TWGs. | Existing government exemptions and national priorities for pregnant women and children were explicitly recognised. Services in these categories were automatically considered under the 'highest priority / existing exemption' classification. |

# 5. Application of Criteria in the Prioritisation Process

The eight criteria were applied across a three-stage prioritisation process:

**Table S4.** Application of the eight decision criteria across the three-stage prioritisation process

| **Stage** | **Description** | **Criteria applied** | **Output** |
| --- | --- | --- | --- |
| **Stage 1** | Automatic inclusion of foundational and exceptionally cost-effective services | Clinical effectiveness (Compendium membership); cost-effectiveness (ACER < USD 100/DALY) | 86 services automatically included; 338 services forwarded to Stage 2 |
| **Stage 2** | Structured TWG deliberation across all eight criteria | All eight criteria applied through expert review and multi-criteria scoring; each TWG covered 8–80 services across 8–12 sessions | 182 services classified as high priority (Priority 1 or Priority 1 conditional); 242 classified as lower priority (Priority 2) |
| **Stage 3** | Fiscal feasibility assessment and scenario modelling | Budget impact (binding constraint); cost-effectiveness (re-applied for ranking); financial risk protection (informs financing structure in Scenario 3) | Three policy scenarios developed: Scenario 1 (182 services, USD 53.76/capita); Scenario 2 (66 services, USD 21.16/capita); Scenario 3 (182 services with differentiated financing, USD 47.74/capita) |

# 6. Limitations of the Criteria Application

Several limitations in the application of criteria should be noted. First, quantitative evidence was unavailable for a substantial proportion of services: 110 of 182 services had no impact estimate (DALYs averted), and many services lacked local or regional cost-effectiveness data. In these cases, TWGs applied qualitative judgement informed by clinical expertise and programme knowledge.

Second, some criteria, particularly financial risk protection and equity, proved difficult to apply consistently across all service types given data constraints. Quantitative thresholds were defined prospectively, but application in practice relied partly on expert consensus.

Third, post-hoc analysis of TWG deliberations revealed that final prioritisation decisions rested mainly on cost-effectiveness, disease burden, and budget impact, with other criteria playing a secondary role. This reflects both the availability of evidence and the fiscal constraints that dominated later stages of the process.

Fourth, some overlap exists between the eight decision criteria, most notably between 'life-threatening conditions' and 'disease severity', reflecting the participatory origin of the criteria set. While TWGs distinguished between these criteria in application, the conceptual overlap may have introduced redundancy in the deliberative weighting of certain services. Future applications may benefit from a formal review of criteria independence before deliberation, or from consolidating overlapping criteria to reduce the burden on participants."
